# mTOR direct crosstalk with STAT5 promotes de novo lipid synthesis and induces hepatocellular carcinoma

**DOI:** 10.1038/s41419-019-1828-2

**Published:** 2019-08-14

**Authors:** Ting Li, Jun Weng, Yue Zhang, Kangyan Liang, Gongbo Fu, Yang Li, Xiaochun Bai, Yi Gao

**Affiliations:** 1 Department of Hepatobiliary Surgery II, Zhujiang Hospital, Southern Medical University, Guangzhou, China; 2 International Cooperation Laboratory on Signal Transduction, Second Military Medical University, Eastern Hepatobiliary Surgery Hospital, Shanghai, China; 3 Institute of Regenerative Medicine, Southern Medical University Zhujiang Hospital, Guangzhou, China; 4 Artificial Organs and Tissue Engineering Centre of Guangdong Province, Guangzhou, China; 5 Department of Cell Biology, School of Basic Medical Sciences, Southern Medical University, Guangzhou, China; 6 State Key Laboratory of Organ Failure Research, Southern Medical University, Guangzhou, China

**Keywords:** Cancer genomics, Cancer metabolism, Cancer models

## Abstract

Hepatocellular carcinoma (HCC) can be the last step of nonalcoholic fatty liver disease (NAFLD) evolution, and the main characteristic of NAFLD is alteration in lipid metabolism. However, the mechanisms of abnormal lipid metabolism in NAFLD and HCC progression are yet to be identified. Here, we demonstrate that liver-specific activation of mTORC1 promoted the expression of lipid synthesis genes and lead to the development of spontaneous HCC. Genetic mouse models developed spontaneous HCC along with increased expressions of SREBP1, ACC1 and FASN. In addition, high levels of p-STAT5 were observed in the livers and particularly evident in the tumor area. And the synthesis of p-STAT5 was increased in patients along with the increase in SREBP1 synthesis in clinical samples. Moreover, mTORC1 interacts with and phosphorylates the STAT5 in hepatocytes. In conclusion, our data suggested that mTORC1 upregulates SREBP1 transcription via crosstalk with the STAT5 pathway which contributes to the NAFLD-related HCC pathogenesis. And the inhibitor of SREBP1 and mTOR may help to prevent HCC in clinical NAFLD patients.

## Introduction

The morbidity and mortality associated with hepatocellular carcinoma (HCC) ranks in the top fine and top three, respectively, among the most common malignant tumors worldwide^1^. An increasing number of reports describe HCC in the setting of obesity and diabetes, two major risk factors for nonalcoholic fatty liver disease (NAFLD). The emerging evidence-linking HCC to noncirrhotic NAFLD indicates that a better understanding of NAFLD-related HCC pathogenesis is required^2^.

Recently, metabolic reprogramming, especially lipid metabolism alterations, has been considered to be the initiating factor of HCC tumor occurrence and progression^3^. A previous study demonstrated that increased expression of lipogenic genes including sterol regulatory element binding protein-1 (SREBP1), fatty acid synthase (FASN), and acetyl-CoA carboxylase was observed in NAFLD patients^4^. SREBP1 is a member of SREBPs family, which is transcription factors that regulate the expression of genes encoding enzymes responsible for the synthesis of fatty acids, triglycerides and cholesterol^5,6^. As the main regulator of hepatic lipogenesis, genetic or pharmacological inhibition of SREBP1 results in human HCC cell lines growth arrest and, decreased cell proliferation^7^.

The mammalian target of rapamycin (mTOR) is a key nutrient-sensing kinase that is aberrantly activated in the liver and other tissues under conditions of obesity^8^. Menon et al. have showed that mice with liver-specific knockout of tuberous sclerosis complex 1 (TSC1), which is an upstream inhibitor of mTORC1, spontaneously developed HCC^9^. mTORC1 activation contributes to regulation of de novo lipogenesis, through upregulating SREBP1 transcription, processing and nucleic accumulation^10^, which have been reported to accelerate HCC development^11^.

It has been shown that hepatocyte-specific ablation of the STAT5 results in progressive steatosis accompanied by elevated liver damage parameters^12,13^. The increase in TG accumulation in the absence of hepatic STAT5 signaling may result from the upregulation of genes involved in hepatic fatty acid uptake and/or de novo synthesis, with SREBP1 as a possible cause^14,15^. However, the precise mechanisms leading to deregulation of SREBP1 signaling upon impaired hepatic STAT5 signaling are not completely understood.

Considering the crucial role of lipid metabolic reprogramming in HCC development, identifying new molecules and pathways that are involved in this process is vital. However, the detailed mechanisms of abnormal lipid metabolism have not yet been comprehensively identified during HCC progression. Genetic mouse models with liver specific knockout of TSC1 (L-TKO mice) are suitable to explore the NAFLD-related HCC pathogenesis because these mice develop spontaneous HCC with a concurrent large accumulation of lipids in hepatocytes. Using this model, we found that the expression of genes related to lipid metabolism was abnormal. Indeed, higher expressions of SREBP1, ACC1, and FASN proteins in L-TKO mice were detected. In addition, we observed relatively high levels of STAT5 and p-STAT5 in the livers of L-TKO mice, which was particularly evident in the tumor. Here, with the in vitro kinase assay, we showed that mTORC1 phosphorylates STAT5 in hepatocytes. Moreover, with the tissue microarrays analysis, we showed that p-STAT5 synthesis was increased in patients along with the increase in SREBP1 synthesis in clinical samples. Taken together, these results demonstrate that mTOR may upregulates SREBP1 transcription via enhanced phosphorylation of STAT5.

## Materials and methods

### Animals

Mice carrying the Tsc1fl allele in the FVB/NJ background have been previous described^16^. L-TKO mice were generated by crossing Tsc1^fl/fl^ (Stock no: 005680) and Alb-Cre (Stock no: 003574) mouse obtained from Jackson Laboratory^17^. Both male and female mice were used. The specificity of recombination was confirmed by PCR using primers flanking the floxed allele. All procedures involving mice was approved by the Ethical Committee for Animal Research of Southern Medical University, Guangzhou, China, and conducted according to the state guidelines from the Ministry of Science and Technology of China. The mice were housed in plastic cages at a controlled temperature of 22 ± 1 °C on a 12-h light/12-h dark cycle with lights on from 06:00 to 18:00 h. Standard rodent chow and water were provided. For rapamycin treatment, L-TKO mice at the age of 5 months were administered rapamycin by oral gavage 5 mg/kg/day until sacrifice at 10 months of age or equal volumes of NS. All mice were sacrificed prior to their daily feeding.

### Histopathology, immunohistochemistry (IHC) and immunofluorescence (IF)

Paraffin-embedded (4–5 μm) livers sections from L-TKO mice and controls were subjected to hematoxylin–eosin (H&E) staining or IHC or IF. For IHC, sections were incubated overnight with antibodies against p-STAT5 (1:100, CST, MA, USA), SREBP1 (1:50, Abcam, Cambridge, UK), FASN (1:100, Abcam), and ACC1 (1:50, Proteintech, Rosemont, USA), followed by HRP-labeled secondary antibodies (Jackson ImmunoResearch, West Grove, PA, USA). For IF, sections were incubated overnight with antibodies against SREBP1 (1:100, Abcam), and cytokeratin 18 (ck18) (1:100, Abcam), followed by Alexa Fluor® 594/488—Conjugated secondary antibodies and DAPI (Life Technologies, MA, USA).

### RNA-seq and bioinformatic analysis

RNA from mouse livers and HepG2 cell lines were isolated using the RNeasy Plus Mini Kit (Qiagen, Hilden, Germany). cDNA library construction and illumine HiSeq4000 sequencing were conducted at Novogene Bioinformatics Institute (Beijing, China). All original microarray data were deposited in the NCBI Gene Expression Omnibus (GEO GSE94687). Bioinformatics analysis was carried out by Novogene bioinformatics and DAVID GO was performed using the online accessible DAVID database (http://david.abcc.ncifcrf.gov).

### Metabolomics

Metabolomic profiling was completed with the assistance of Shanghai Biotree Biotech Co. Ltd. All data were analyzed by gas chromatography time-of-flight mass spectrometry.

### Gene expression analysis

RNA isolated was described above, followed by cDNA generation use PrimeScrip RT Master Mix (TaKaRa, Tokyo, Japan). Gene expression of SREBP1, ACC1, FASN, Mup10, Att3, Ddit3, and Apoa4 was measured by the StepOnePlus Real-Time PCR System (Life) and ChamQ SYBR Color qPCR Master Mix (Vazyme Biotech, Nanjing, China), β-actin was measured as a reference gene. Primer pair sequences were as follows: Mup10:

forward: 5′-GAAGAGATGAAGAGTGCTCCGAA-3′,

reverse: 5′- TGTGCAAACCTTTCCTTGATGTC-3′;

Atf3:

forward: 5′-GAGGATTTTGCTAACCTGACACC-3′,

reverse: 5′-TTGACGGTAACTGACTCCAGC-3′;

Ddit3:

forward: 5′-CTGGAAGCCTGGTATGAGGAT-3′,

reverse: 5′-CAGGGTCAAGAGTAGTGAAGGT-3′;

Apoa4:

forward: 5′-CCAATGTGGTGTGGGATTACTT-3′,

reverse: 5′-AGTGACATCCGTCTTCTGAAAC-3′;

SREBP1:

forward: 5′-AATCACTGAAGGACCTGGTGT-3′,

reverse: 5′-CTCAGAGTCACTACCACCACTG-3′;

FASN:

forward: 5′-GGAGGTGGTGATAGCCGGTAT-3′,

reverse: 5′-TGGGTAATCCATAGAGCCCAG-3′;

ACC1:

forward: 5′-TGAATGTGAGAATCCAAGTGAGC-3′,

reverse: 5′-GGTCTGTTTAACAAAGTCAGGGA-3′;

β-actin:

forward: 5′-CCTGAGGCTCTTTTCCAGCC-3′,

reverse: 5′-TAGAGGTCTTTACGGATGTCAACGT-3′.

### Western blotting

Liver tissue or cultured cells were lysed in RIPA buffer containing 50 mM Tris-HCl pH 8, 150 mM NaCl, 1% Triton X-100, 0.1% sodium deoxycholate, 0.1% Sodium dodecyl sulfate (SDS), phosphatase inhibitors and protease inhibitor cocktail (Roche). Solubilized proteins were collected by centrifugation and quantified using a BCA protein assay kit (Thermo Scientific). Proteins were separated on SDS-PAGE gels and transferred to polyvinylidene difluoride membranes (Bio-Rad, CA, USA) for blotting with antibodies. Primary antibodies: AKT, p-AKT, S6K, PS6K, p-4EBP1, p-STAT5, STAT5 (CST), SREBP1, FASN (Abcam), ACC1 (Proteintech), and β-actin (Sigma-Aldrich, Darmstadt, Germany).

### Coimmunoprecipitation

Liver tissues were lysed with lysis buffer containing 20 mM Tris-HCl pH 8, 150 mM NaCl, 0.5% Nonidet P-40, 2 mM EDTA, 0.5 mM DTT, 1 mM NaF, 1 mM PMSF and 1× protease inhibitor cocktail. Lysates were clarified by centrifugation at 10,000 *g* for 10 min at 4 °C and then incubated with anti-mTOR and anti-STAT5 antibodies or control IgG followed by precipitation with protein G conjugated agarose beads. Beads were washed four times with lysis buffer and boiled with 1× SDS sample for 5 min. Then analysed by western blotting.

### Cell culture and treatments

HepG2 cells were cultured in DMEM medium (Gibco) containing 10% FBS (Gibco) with 5% CO_2_ at 37 °C. Cells were incubated with IN-1 to inhibit p-STAT5 for 36 h. For gene knockdown or overexpression, HepG2 cells were grown to confluence and transfected with siRNA or plasimid using lipo3000 (Life Technologies) for 48 h and protein and mRNA collected for protein extraction and RNA expression analysis. For the luciferase assay, HepG2 cells were transfected with siRNA or plasimid using lipo3000 for 24 h, luciferase production was determined by the Dual Luciferase Reporter Assay System (Promega, WI, USA), and luminescence acquired by an EnVision microplate reader (PerkinElmer). For induced steatosis, HepG2 cells were cultured in six-well plates at 5·10^5^ cells/well. The cells were treated with 0.2 mM oleic acid and 0.4 mM palmitic acid solution for 24 h and used for laboratory analyses. Oleic acid and palmitic acid (Sigma-Aldrich) were dissolved at a concentration of 10 mM in sodium hydroxide solution (0.1 M NaOH), which contained 10% fatty acid-free BSA (Sigma-Aldrich), respectively.

### In vitro kinase assay

In vitro kinase assay was performed as described previously^18–20^. After treatment, cells were washed twice with ice-cold PBS and lysed in ice-cold buffer containing 40 mM HEPES (pH 7.4), 2 mM EDTA, 10 mM pyro-phosphate, 10 mM glycerophosphate, 0.3 % CHAPS, one tablet of EDTA-free protease inhibitors (Roche) per 25 ml. Then, the cell lysates were isolated by centrifugation at 12,000 rpm for 10 min in a microcentrifuge, and incubated with anti-mTOR antibody for 2 h at 4 °C, followed by addition of 30 μl of 50% slurry of protein G Sepharose beads for an additional 1 h. Immunoprecipitates were then washed four times with lysis buffer and once with kinase buffer [25 mM HEPES (pH 7.4), 50 mM KCl, 10 mM MgCl2, 250 μM ATP]. In all 0.4 μg of recombinant GST-tagged full-length STAT5 peptide (Creative BioMart, NY, USA) was added to 30 μl kinase buffer. Reactions were stopped by the addition of 30 μl SDS sample buffer and boiling for 10 min and analyzed by immunoblotting. All experiments are representative of three independent experiments.

## Results

### Liver-specific activation of mTORC1 is associated with hepatosteatosis and spontaneous HCC development

**Fig. 1:**
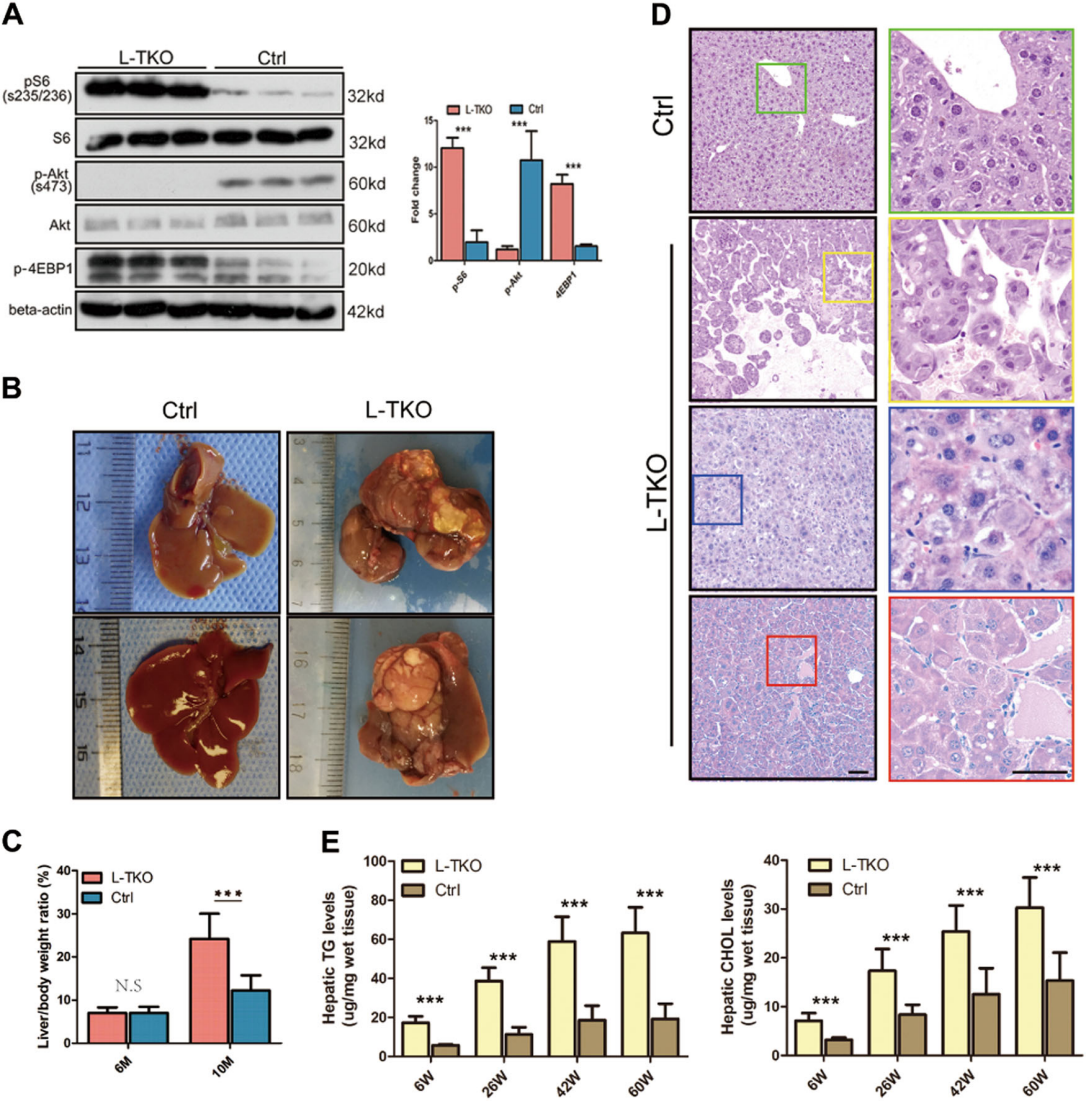
L-TKO mice spontaneously developed HCC with lipid accumulation. L-TKO mice generated by crossing TSC1fl/fl mice with Alb-Cre mice were provided standard rodent chow for 6–10 months. **a** Liver tissues were lysed and mTORC1 signal activity was determined by the high levels of pS6 and p-4EBP1 and the low level of p-Akt. **b** L-TKO mice spontaneously developed HCC and randomly distributed in liver lobes by 10 months. **c** L-TKO mice exhibited increased liver/body weight ratio by 10 months. **d** H&E staining showed two histopathological types, clear cell (yellow) and trabecular (blue and red). **e** Hepatic TG and CHOL from L-TKO and control mice were determined. Western blots were quantified. *n* ≥ 6, ***P < 0.001

The knockdown efficiency of TSC1 gene in L-TKO mice was confirmed by polymerase chain reaction (PCR) technology (data not shown). L-TKO mice displayed constitutively active mTORC1 signaling in the liver, as indicated by the high levels of S6 kinase phosphorylation (pS6) and eukaryotic initiation factor 4E-binding protein 1 (4E-BP1). Conversely, these mice exhibited low levels of the p-AKT which were comparable to control (TSC1^fl/fl^) littermates (Fig. 1a).

As expected, there were no detectable tumors until 10 months, an average of three tumors without capsules were randomly distributed in all liver lobes was detected in L-TKO mice, with no macroscopic tumors observed in the control littermates (Fig. 1b). Changes in the liver/body weight ratio was also observed in L-TKO mice an qo months age (Fig. 1c). Histopathological analysis revealed two major types of tumors, clear cell and trabecular (Fig. 1d). To further explore whether there was hepatic steatosis in L-TKO mice, we detected the hepatic triglyceride (TG) and cholesterol (CHOL) levels. Consistent with our conjecture, hepatic TG and CHOL levels were increased in L-TKO mice (Fig. 1e). Collectively, our results indicate that dysregulation of the hepatic mTOR pathway is involved in hepatic steatosis and HCC.

### Liver-specific activation of mTORC1 promotes expression of lipid synthesis genes

**Fig. 2:**
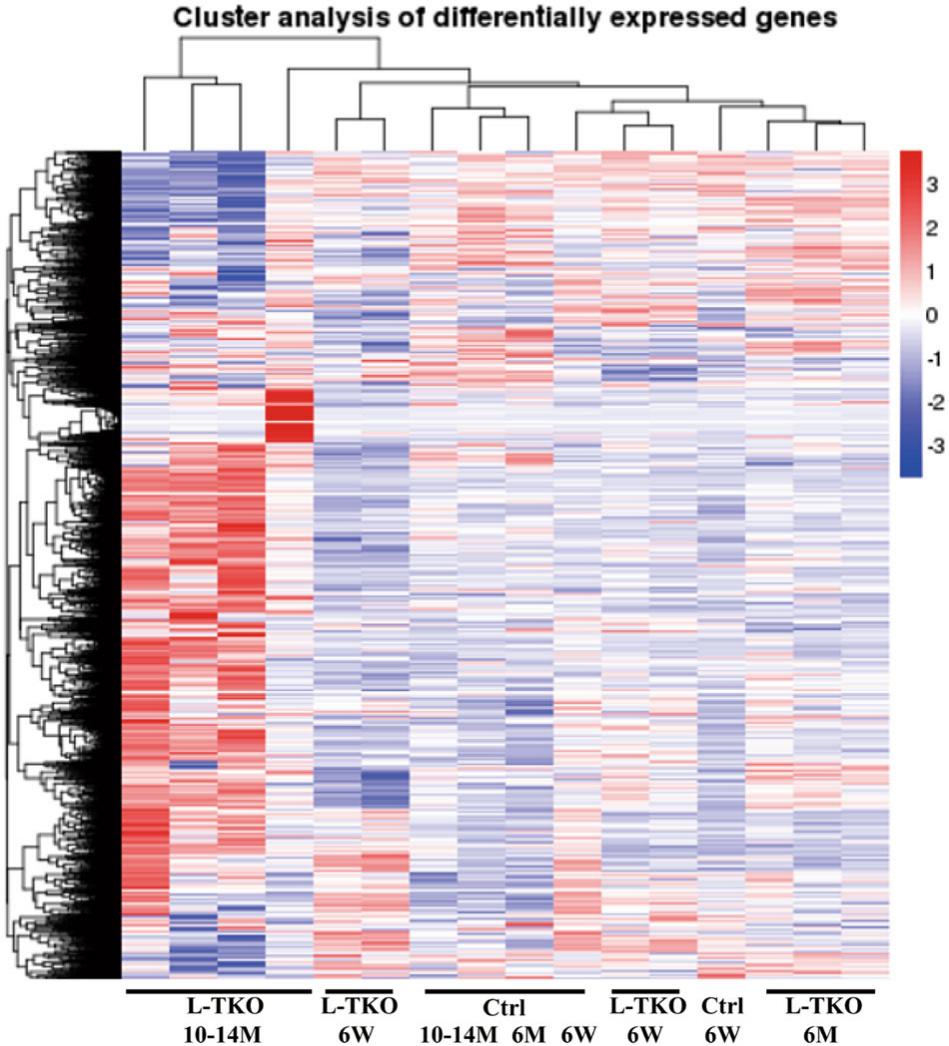
Hierarchical clustering of transcripts that are differentially expressed between livers in L-TKO mice and controls. The colors in the map reflect a log2 scale. The brightness of blue and red represents the degree to which expression is respectively lower or higher in the L-TKO mice to the controls. Peak areas of each gene were normalized by total ion count and the map displays the average value for each gene

**Fig. 3:**
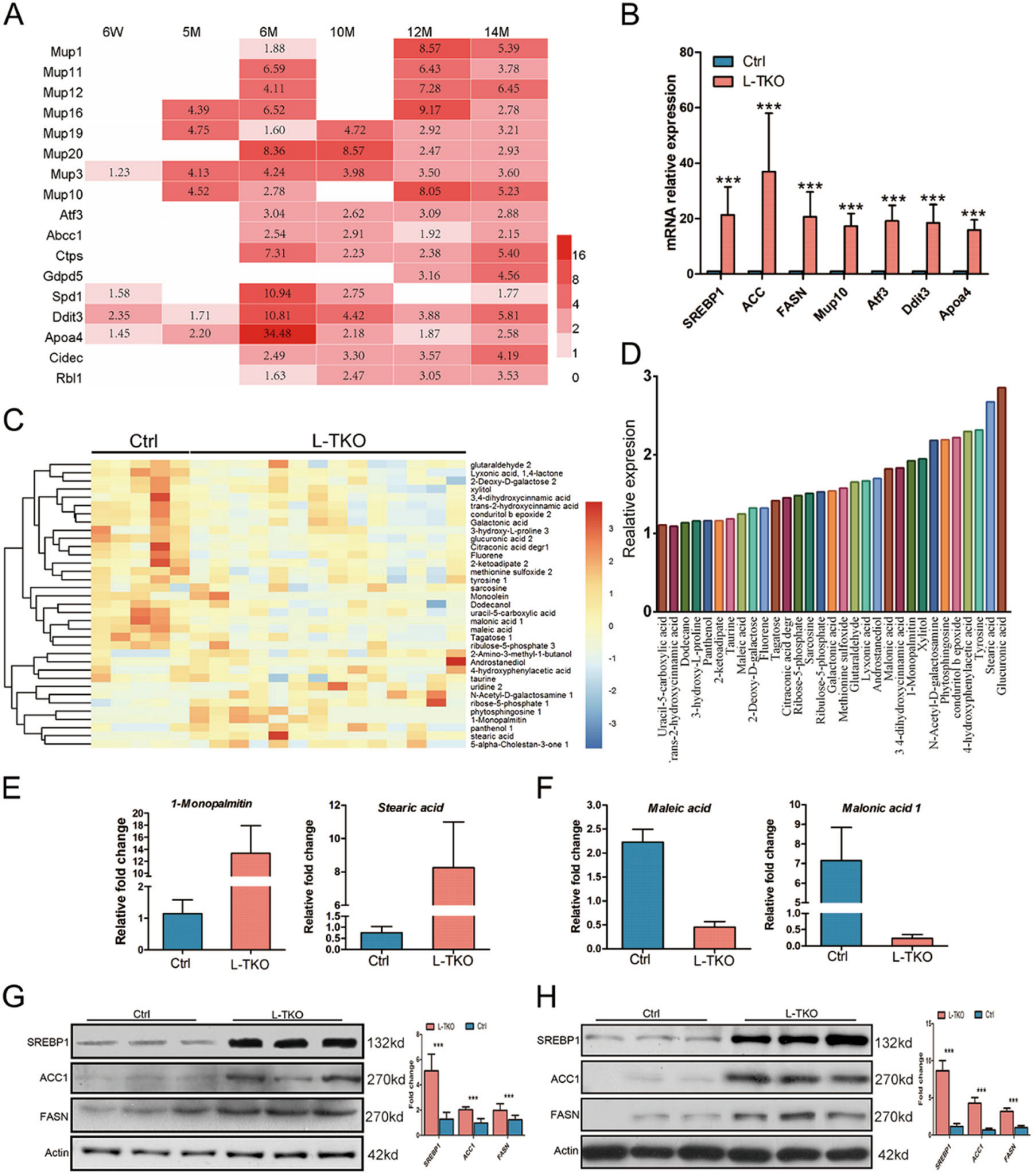
Increased lipid synthesis in L-TKO mice. **a** RNA-seq showed significantly upregulated in biological process of lipid synthesis genes based on the ratio of L-TKO/control mice of indicated ages (*n* ≥ 4). **b** Related hepatic mRNA (qPCR) from 10-month-old L-TKO and control mice (*n* = 7, ***P <0.001). **c**, **d** Metabolome analysis showed increased steatosis in 10-month-old L-TKO mice compared with 6-month-old L-TKO mice (*n* = 10). **e**, **f** Increased fatty acids and decreased glucose were observed in 10-month-old L-TKO compared with controls (*n* = 10). **g**, **h** SREBP1(m), ACC1, and FASN proteins were assessed by western blot at 6-month-old (**g**) and 10-month-old (**h**) L-TKO mice and controls. Western blots were quantified. *n* ≥ 6, ***P <0.001

RNA-seq analysis was performed, and pathway analysis revealed alterations in many metabolic related pathways that influence various biological processes, such as lipid synthesis (Fig. 2). Further gene ontology (GO) enrichment analysis revealed that differentially expressed genes were significantly enriched in lipid synthesis genes, including mups, Atf3, and Ddit3 (Fig. 3a). Considering that SREBP1 regulates lipid synthesis, we tested related genes by qRT-PCR, and the expressions of these genes were upregulated (Fig. 3b).

We futher checked whether increased steatosis occurred. Specifically, the metabolome was investigated (Fig. 3c, d). Consistent with RNA-seq, metabolomic changes increased fatty acids (Fig. 3e), and decreased glucose metabolites (Fig. 3f). Western blotting also revealed higher expression of SREBP1, ACC1, and FASN in L-TKO livers (Fig. 3g, h). These findings collectively suggest that specific metabolic pathway changes might contribute to tumor progression in the L-TKO mice, a finding which is commonly observed in human NAFLD-associated HCC.

### mTORC1 promotes de novo lipid synthesis

**Fig. 4:**
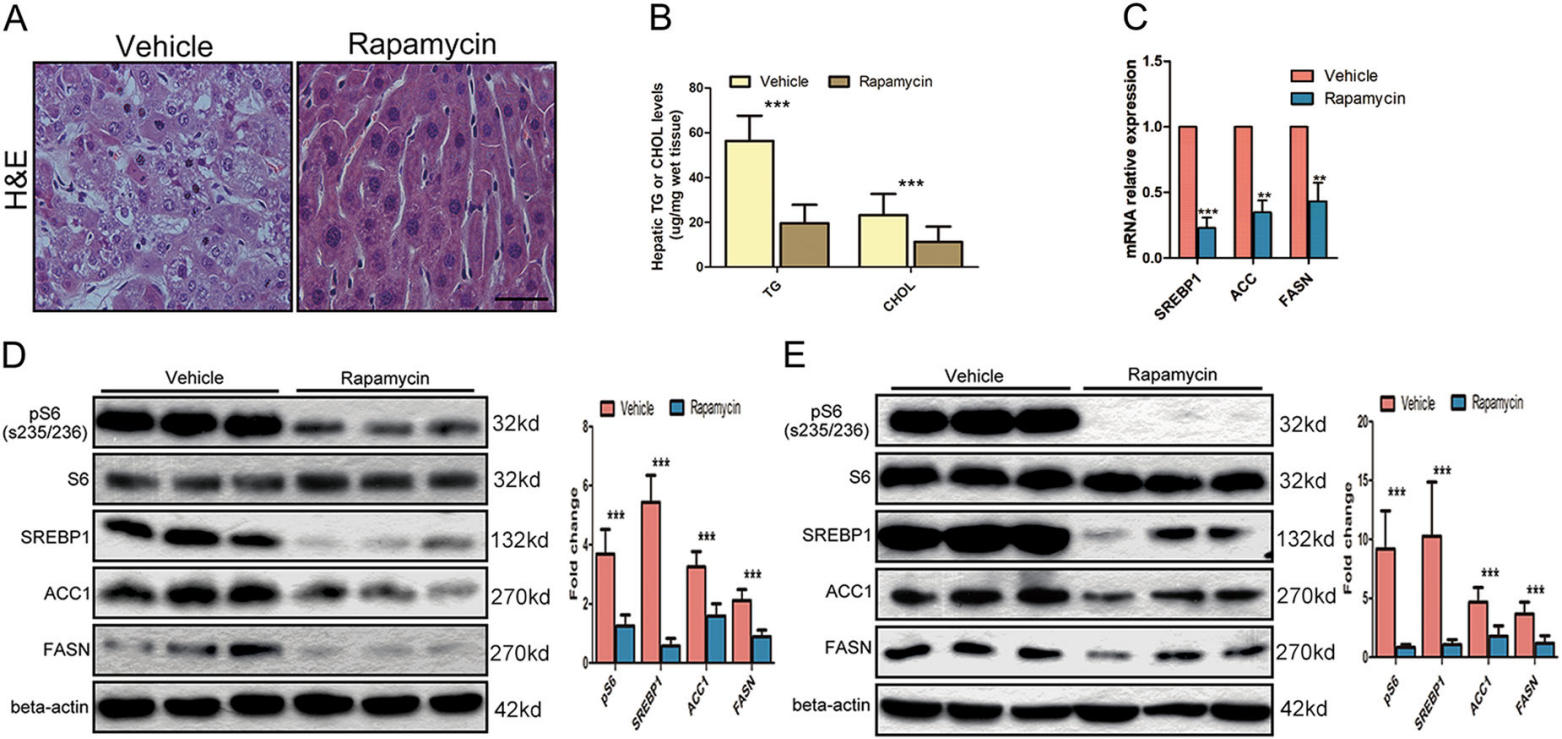
Rapamycin inhibits lipid synthesis and tumorigenesis. L-TKO mice were administrated with rapamycin (2.5 mg kg^−1^ d^−1^) or drug vehicle for 5 months. **a** H&E staining showed that rapamycin-treated L-TKO mice developed no tumor. **b** Hepatic TG and CHOL were determined. **c** Related hepatic mRNA (qPCR) from rapamycin or vehicle treated 10-month-old L-TKO mice (*n* = 6, **P < 0.01, ***P < 0.001). **d** Liver tissues were lysed and mTORC1 signal activity was determined by the high levels of pS6, lipid synthesis inhibition was determined by the low levels of SREBP1(m), ACC1 and FASN. **e** HepG2 cells treated with rapamycin (1.0 × 10^−7^ m) or drug vehicle for 24 h, and pS6, SREBP1(m), ACC1 and FASN were determined. Western blots were quantified. *n* ≥ 6, ***P < 0.001

The above findings suggest that a hyperactive mTORC1 pathway promotes de novo lipid synthesis. To further confirm whether tumorigenesis is indeed mTORC1 dependent, 22-week-old L-TKO mice were treated with the mTORC1 inhibitor rapamycin or with an equal volume of normal saline (NS) for 22 weeks. Rapamycin-treated L-TKO mice showed reduced mTORC1 signaling, with no liver tumor development (Fig. 4a), indicating that tumorigenesis was mTORC1 dependent. Moreover, hepatic lipid accumulation was reversed in rapamycin-treated mice and reduced mRNA and protein levels of SREBP1, ACC1 and FASN (Fig. 4b–d). We also tested at the cellular level, HepG2, a human HCC cell line, was treated with rapamycin for 24 h. Rapamycin-treated HepG2 showed reduced mTORC1 signaling, and reduced expression of SREBP1, ACC1, and FASN proteins (Fig. 4e). These results suggested that SREBP1 contributes to HCC development by regulating protein expression of ACC1 and FASN through the mTORC1 pathway.

### mTORC1 phosphorylation of STAT5 is linked to hepatosteatosis and HCC in L-TKO mice

**Fig. 5:**
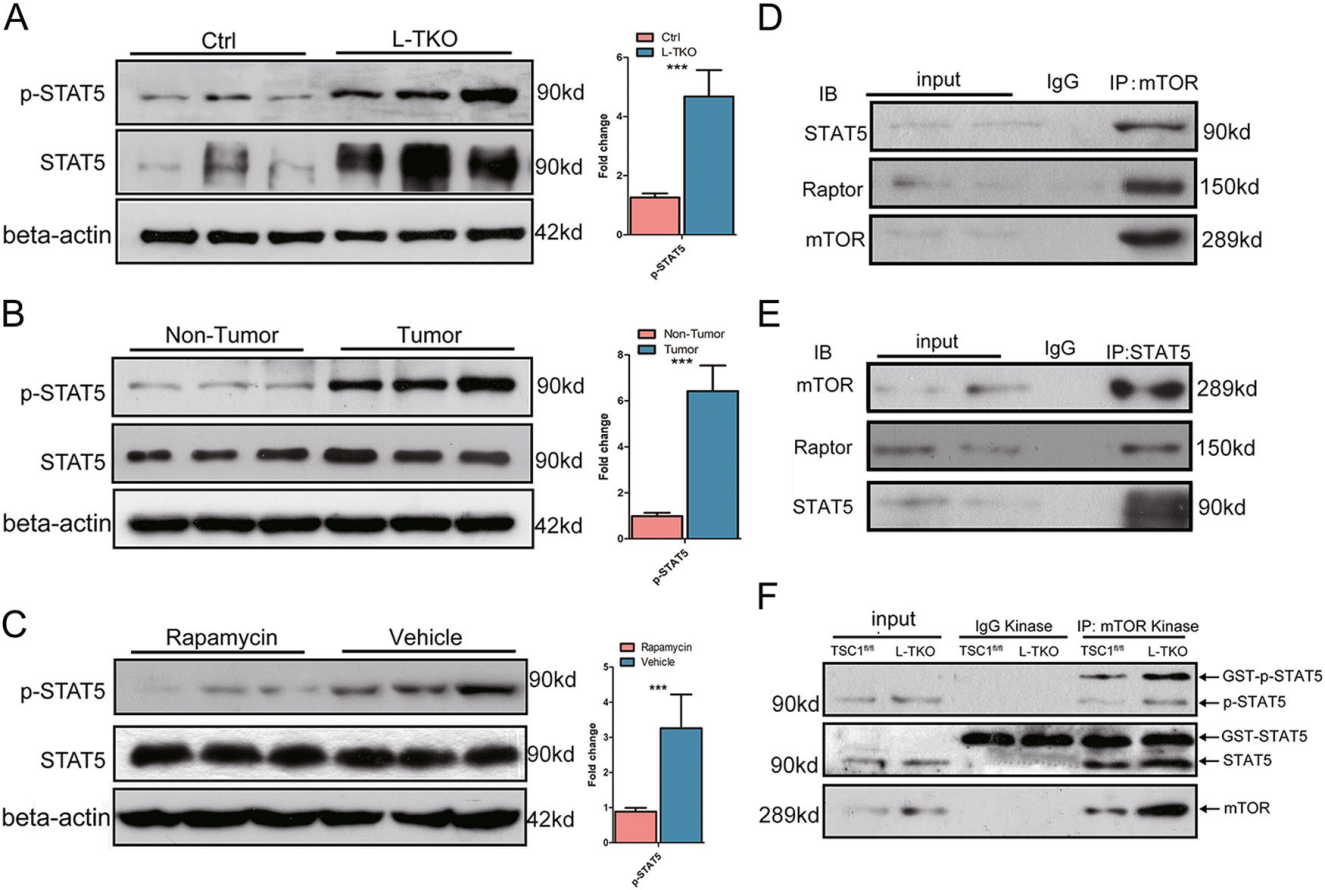
mTOR interacts with and phosphorylates the STAT5. Hepatic p-STAT5 was determined by western blots in L-TKO mice and control littermates (**a**), and in tumor and nontumor areas of L-TKO mice (**b**). **c** Liver tissues were lysed from rapamycin or vehicle treated 10-month-old L-TKO mice, and p-STAT5 was determined by western blots (*n* = 6). Western blots were quantified. ***P < 0.001. **d** HepG2 cells were immunoprecipitated with anti-mTOR antibody and the amounts of Raptor, and STAT5 co-purified with mTOR were determined by western blot. **e** HepG2 cells were immunoprecipitated with anti-STAT5 antibody and the amounts of mTOR and Raptor co-purified with STAT5 were determined by western blot. **f** Cultured hepatocytes cells from Ctrl and L-TKO mice were immunoprecipitated with anti-mTOR antibody and the precipitated mTOR was assayed for kinase activity against recombinant GST-tagged full-length STAT5

It has been showed that expression of STAT5 alters lipid metabolism and progressive steatosis^21^. Relatively high p-STAT5 levels were detected in the livers of L-TKO mice, especially in tumors (Fig. 5a, b). Rapamycin-treated L-TKO livers showed reduced p-STAT5 expression (Fig. 5c). We therefore proposed that there was interaction between mTORC1 and STAT pathways. The results of immunoprecipitation analysis revealed that mTOR and Raptor interacts with STAT5, definitely, in HepG2 cell lines (Fig. 5d, e). Next, to detect whether mTOR phosphorylates STAT5 in hepatocytes, we performed in vitro kinase assays in the hepatocytes from control and L-TKO mice, and confirmed that mTORC1 does phosphorylate STAT5 in hepatocytes. Moreover, the activation of mTORC1 enhanced the expression of p-STAT5 (Fig. 5f), indicating an interaction between mTORC1 and the STAT pathway.

### p-STAT5 interacts with SREBP1 and promotes its transcriptional activity

**Fig. 6:**
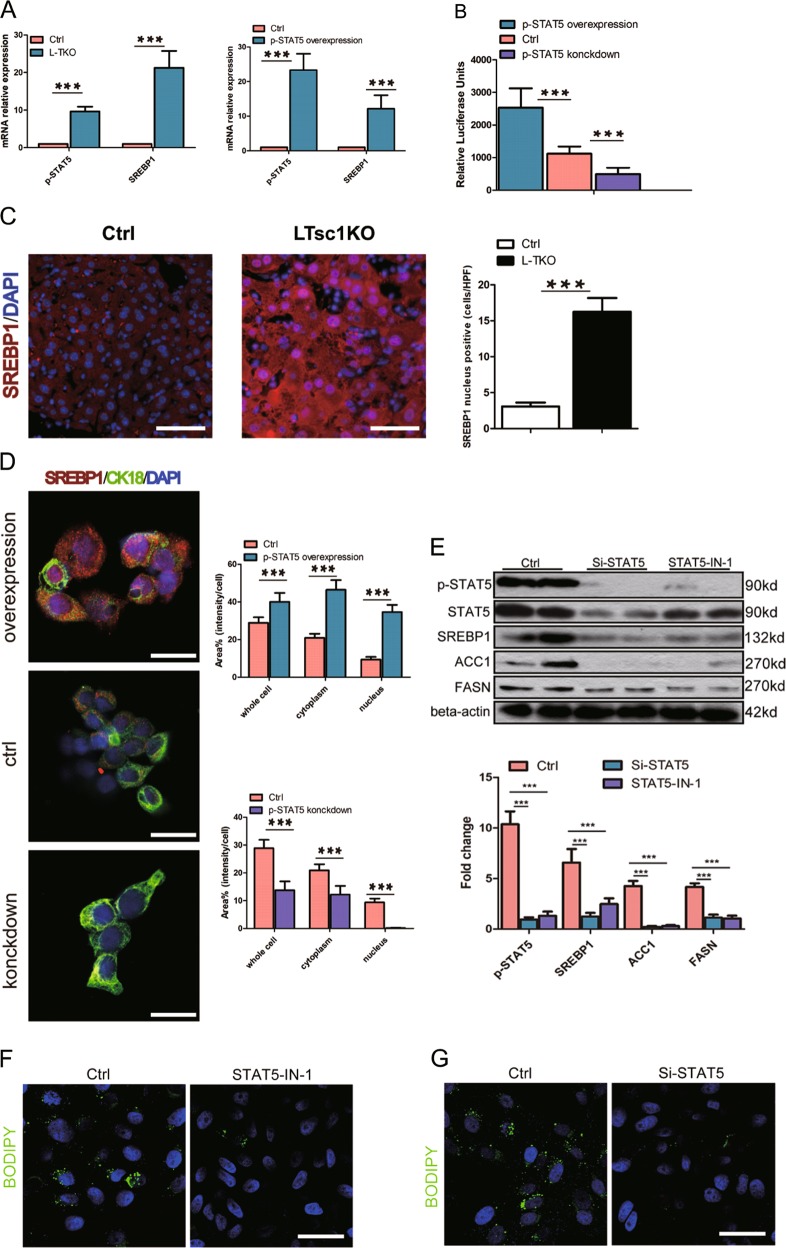
p-STAT5 interacts with SREBP1 and promotes its transcriptional activity. **a** Related hepatic mRNA (qPCR) from 10-month-old L-TKO and control mice (left, *n* = 5, ***P < 0.001), and p-STAT5-plasmid treated HepG2 cells (right, *n* = 6, ***P < 0.001). **b** Relative luciferase activity was detected after HepG2 cells were co-transfected with SREBP1 promoter and p-STAT5-siRNA or p-STAT5-plasmid (*n* = 6, ***P < 0.001). **c** Liver tissues were collected and stained with anti-SREBP1 showed significantly accumulated both in the cytoplasm and the nuclear componets in L-TKO mice (*n* = 4, ***P < 0.001). **d** HepG2 cells were treated with p-STAT5-siRNA or p-STAT5-plasmid to downregulate or upregulate p-STAT5, which affected the nuclear distribution of SREBP1 (*n* = 6, ***P < 0.001). **e** HepG2 cells were treated with siRNA or inhibitor IN-1 to downregulate p-STAT5, which reduced the expression of SREBP1 (m), ACC1 and FASN. Western blots were quantified. *n* = 4, ***P < 0.001. HepG2 cells were treated with inhibitor IN-1(**f**) or siRNA (**g**) to inhibit p-STAT5 in advance, which decreased lipid accumulation induced by oleic acid and palmitic acid, confirmed by BODIPY493/503 staining

Hepatic p-STAT5 and SREBP1 increased simultaneously in L-TKO mice, SREBP1 is known to be downstream of mTORC1^22,23^, then we assumed that SREBP1 is also downstream of p-STAT5. Consistent with protein expression levels, mRNA levels of p-STAT5 and SREBP1 were also increased in L-TKO liver. Moreover, in p-STAT5 overexpressed HepG2 cells, the mRNA level of SREBP1 also increased (Fig. 6a). To examine whether p-STAT5 affects the activity of SREBP1 promoter, p-STAT5 siRNA, or plasmid was transfected and luciferase assays were performed. Downregulation of p-STAT5 decreased, whereas overexpression of p-STAT5 increased, the activation of the SREBP1 promoter (Fig. 6b). Immediately, we examined whether p-STAT5 had an effect on the nuclear translocation of SREBP1. SREBP1 significantly accumulated both in the cytoplasm and the nuclear componets in L-TKO mice (Fig. 6c). Moreover, p-STAT5 overexpression in HepG2 cells significantly promoted the nuclear distribution of SREBP1. On the other hand, p-STAT5 knockdown in HepG2 cells caused an obvious reduction of SREBP1 in the nuclear components (Fig. 6d). These results suggested that p-STAT5 could affect the expression and the nuclear distribution of SREBP1, thus regulating the biological functions of this protein. Next, HepG2 cells were treated with IN-1 or siRNA to inhibit p-STAT5. As expected, protein levels of SREBP1, ACC1 and FASN were decreased (Fig. 6e). Similarly, decreased lipid accumulation in oleic acid and palmitic acid-treated HepG2 cells, which treated with IN-1 or siRNA to inhibit p-STAT5 for 48 h in advance, was confirmed by BODIPY493/503 staining (Fig. 6f, g).

Collectively, these results showed that hepatic deletion of TSC1 induces hepatosteatosis and HCC, which is dependent on the nuclear translocation activation of SREBP1, likely through the crosstalk between mTORC1 and the STAT pathway.

### STAT5 and lipogenesis are upregulated in human hepatosteatosis progressing to HCC

**Fig. 7:**
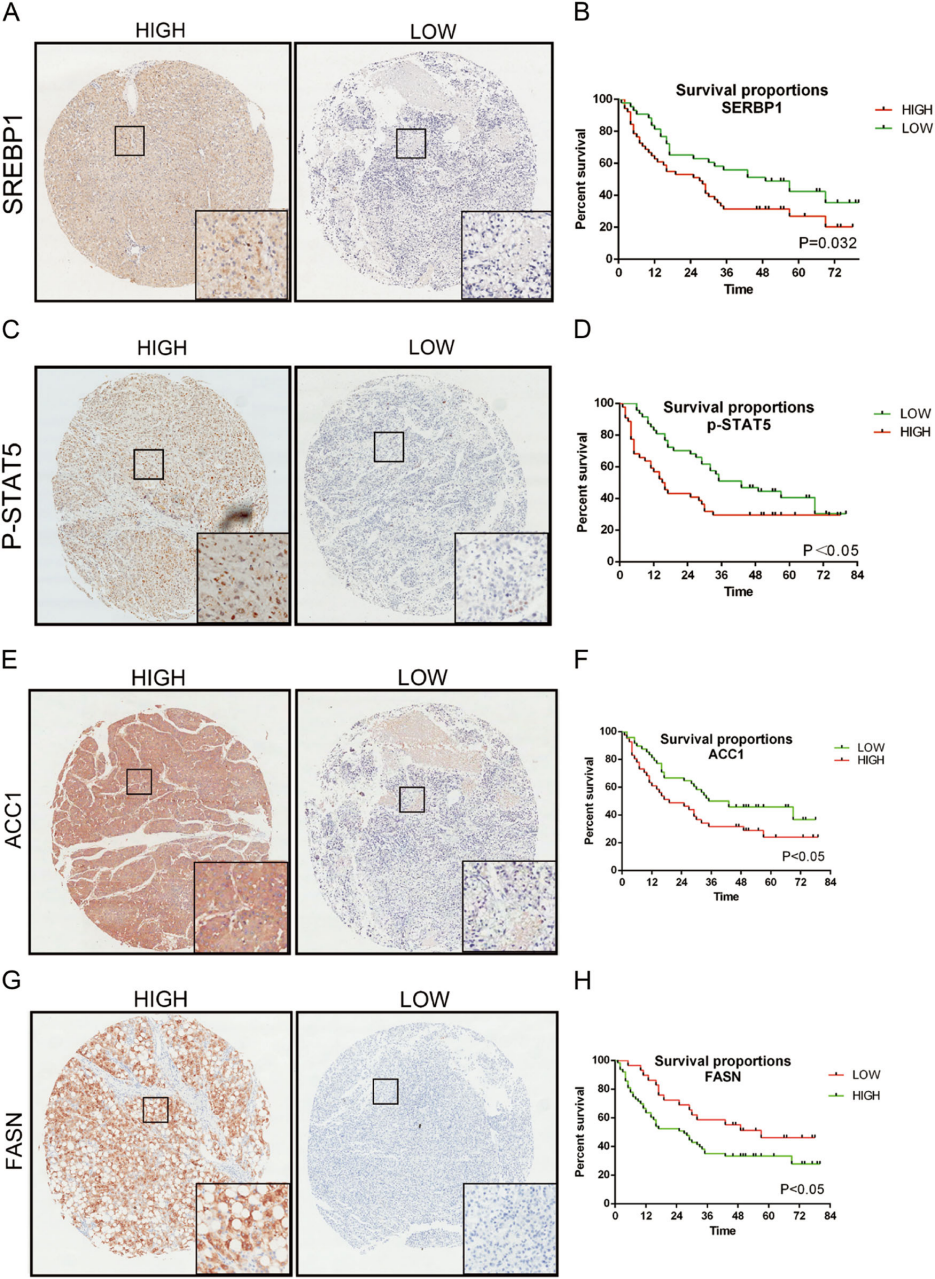
SERBP1 and p-STAT5 levels predicted prognosis of HCC. Eighty-three pairs HCC tissue samples were collected and stained with anti-SREBP1 (**a**), p-STAT5 (**c**), ACC1 (**e**), or FASN (**g**). Strong (up), weak (down). Association between SREBP1 (**b**), p-STAT5 (**d**), ACC1 (**f**), or FASN (**h**) expression levels and overall survival rate in patients with HCC were analyzed by Kaplan–Meier’s analysis

L-TKO mice displayed hepatocyte lipid accumulation progressing to HCC, which was similar to NAFLD to HCC patients in the clinic. Tissue microarrays analysis revealed that the p-STAT5 and the SREBP1 synthesis was increased (Fig. 7a, c), along with the increased of ACC1 and FASN synthesis (Fig. 7e, g). While the survival rates of patients with high expression of STAT5, SREBP1, ACC1 or FASN was relatively low (Fig. 7b, d, f, h). Thus, mTOCR1 may promote the SREBP1 synthesis and the phosphorylation of STAT5, which may contribute to HCC development in patients with NAFLD. Taken together, our results suggest that use of a SREBP1 inhibitor or rapamycin may help to prevent HCC in clinical NAFLD patients.

## Discussion

Previous study demonstrated that aberrant activation is a dominant oncogenic event in human HCC, and de novo lipogenesis is believed to be involved in oncogenesis^24^. NAFLD is a disease in which patients have abnormal lipid metabolism. The emerging evidence linking HCC in NAFLD suggest a better understanding of NAFLD-related HCC pathogenesis required^2^. Upregulation of FASN, ACC1 levels has been showed in a small HCC collection^25^. SREBP1 expression has been found to inversely correlate with patients’ prognosis^26^. However, there are few genetic mouse model for NAFLD-related HCC.

mTORC1 is a key component in a signaling network that regulates metabolism. So we examined tumorigenesis in an mTOR-dependent HCC mouse model (L-TKO mice). We report that mTORC1 promotes tumorigenesis through crosstalk with p-STAT5 via de novo lipogenesis. And similar to patients who develop NAFLD that progress to HCC, L-TKO mice were characterized by a large accumulation of lipids in hepatocytes. Normal tissues often utilize circulating lipids, while more than 90% of fatty acids are produced from de novo synthesis in tumors cells during their rapid growth and proliferation^27^. Thus, we suggest that mTORC1 activation contributes to NAFLD-related HCC.

RNA-seq analysis confirmed that the expression of genes related to lipid metabolism was abnormal in L-TKO mice. And significant changes in SREBP1, FASN, and ACC1 expressions were observed. Guri et al.^28^ thought that mTORC2 regulates de novo lipogenesis to promote tumorigenesis. While, previous studies have showed that mTORC1 activation contributes to regulation of de novo lipogenesis, through upregulating SREBP1 transcription, processing and nucleic accumulation^10^. Considering, mTORC1 also regulates lipid de novo synthesis, especially in L-TKO mice.

We observed relatively high p-STAT5 levels particularly at the tumor sites in the L-TKO mice. Moreover, TG accumulation in the absence of hepatic STAT5 signaling may result in the upregulation of SREBP1^14,15^. These findings suggest that mTORC1 may regulate SREBP1 via phosphorylation levels of STAT5. And our data revealed that mTORC1 interacts with STAT5 directly and enhanced the phosphorylation of STAT5. Moreover, SREBP1 has been found to activate the fatty acid pathway in human HCC cell lines. Thus, we assumed that mTORC1 promotes HCC through SREBP1 and crosstallk with p-STAT5.

How does p-STAT5 affect the biological behavior of SREBP1? First, increased expression of SREBP1 was observed in L-TKO mice, accompanied by an increase in the expression of p-STAT5. Second, p-STAT5 knockdown HepG2 cells was associated with decreased lipogenesis and downregulation of lipogenic proteins, and forced overexpression of p-STAT5 resulted in induction of lipid biosynthesis and upregulation of lipogenic proteins. Modulation of SREBP1, FASN and ACC1 was able to significantly affect STAT5 activation. Using the luciferase assay, we demonstrated that p-STAT5 increase the activity of SREBP1. Third, we demonstrated that p-STAT5 also could affect the nuclear distribution of SREBP1.

Furthermore, elevated expression of p-STAT5 and SREBP1 also occurred in NAFLD-related HCC patients, along with poor prognosis. This data strongly suggests that mTOR upregulates SREBP1 transcription via enhanced phosphorylation of STAT5. A previous study showed that positive expression of SREBP1 was correlated with poor clinicopathological parameters, which was an independent prognostic factor in HCC^29^. Combined with our findings, increase in p-STAT5 expression may predict a poor clinical prognosis.

Rapamycin reduced hepatosteatosis was observed in human patients^30^, in other words, mTORC1 activation stimulated lipid synthesis. Therefore, we revealed a previously unappreciated role for dysregulated mTORC1 signaling in promoting cancer-initiating events via activation of STAT5, wherein mTORC1 upregulates SREBP1 transcription via crosstalk with the STAT5 pathway, which contributes to the NAFLD-related HCC pathogenesis.

In summary, we revealed a previously unappreciated role for mTORC1 upregulates SREBP1 transcription via crosstalk with the STAT5 pathway which contributes to the NAFLD-related HCC pathogenesis. Overall, we demonstrate that mTORC1 upregulates SREBP1 by directly enhancing phosphorylation of STAT5. In light of our findings, we suggest that SREBP1 and p-STAT5 are promising prognostic predictor for patients with HCC, and the mTOR-p-STAT5-SREBP1 axis is a potential therapeutic target for HCC treatment.
